# Quanduzhong capsules for the treatment of grade 1 hypertension patients with low-to-moderate risk: A multicenter, randomized, double-blind, placebo-controlled clinical trial

**DOI:** 10.3389/fphar.2022.1014410

**Published:** 2023-01-10

**Authors:** Xuan Xu, Wende Tian, Wenhui Duan, Chaoxin Pan, Mingjian Huang, Qinggao Wang, Qinghua Yang, Zhihao Wen, Yu Tang, Yao Xiong, Zhiyun Zhu, Yuanyuan Liu, Dan Wei, Wenqiang Qi, Xiaochao Ouyang, Shaozhen Ying, Xiaohua Wang, Zhigang Zhou, Xiaofeng Li, Yu Cui, Shuyin Yang, Hao Xu

**Affiliations:** ^1^ Xiyuan Hospital, China Academy of Chinese Medical Sciences, Beijing, China; ^2^ National Clinical Research Center for Chinese Medicine Cardiology, Xiyuan Hospital, China Academy of Chinese Medical Sciences, Beijing, China; ^3^ The Eighth Hospital of Baotou, Baotou, China; ^4^ Graduate School, China Academy of Chinese Medical Sciences, Beijing, China; ^5^ The First Affiliated Hospital of Guangxi University of Chinese Medicine, Guangxi, China; ^6^ Jiangxi Provincial People’s Hospital, Nanchang, Jiangxi Province, China; ^7^ Jiangxi Puzheng Pharmaceutical Co, Ltd., Jiangxi, China

**Keywords:** quanduzhong capsule, traditional Chinese medicine, grade 1 hypertension, low-to-moderate risk, randomized controlled trial

## Abstract

**Background:** Duzhong [DZ (*Eucommia ulmoides* Oliv.)] is regarded as a traditional Chinese medicine with a history dating back more than 2000 years. This herb is considered a nourishing herb in China and is commonly used as a tonic to strengthen muscles and bones, nourish the kidneys and liver, and soothe miscarriages. Moreover, there is evidence that DZ is capable of regulating blood pressure (BP), and several compounds isolated from DZ have been shown to have a BP-lowering effect. Quanduzhong capsules contain an extract of DZ [*Eucommia ulmoides* Oliv. (Eucommiaceae; Eucommiae cortex)] that is effective in treating hypertension. This multicenter, randomized, double-blind, placebo-controlled clinical trial sought to evaluate the clinical efficacy of Quanduzhong capsules in the treatment of low-to-moderate risk grade 1 hypertension patients.

**Materials and methods:** A total of 60 patients from 3 centers with documented low-to-moderate risk grade 1 hypertension were randomly assigned in a 1:1 ratio to the test group or the control group. After a 1 week lead-in period using sham Quanduzhong capsules, all patients who met the entry criteria (29 cases in the test group and 29 cases in the control group) entered the 4 week test period. The test group took Quanduzhong capsules, and the control group continued to take sham Quanduzhong capsules. The primary endpoints [24-h mean systolic blood pressure (SBP) and diastolic blood pressure (DBP) determined *via* 24-h ambulatory blood pressure monitoring (ABPM); office SBP and DBP] and secondary endpoints [mean arterial pressure; mean pulse; daytime mean SBP and DBP; nocturnal mean SBP and DBP; SBP and DBP load; area under the blood pressure (BP) curve; morning peak BP; early morning SBP and DBP; smoothness index of SBP and DBP; 24 h BP mean coefficient of variation (CV); percentage of patients with circadian restoration in ABPM; home BP; quality of life evaluated by WHO Quality of Life-BREF questionnaire; grading and quantitative evaluation of hypertension symptoms; values of plasmatic renin activity, angiotensin II, aldosterone, β-2 microglobulin and homocysteine] were assessed following the treatment. Drug-related adverse events and adverse drug reactions were also compared.

**Results:** After a 4 week test period, a significant difference in the DBP CV between the two groups was observed (−2.49 ± 4.32 vs. 0.76 ± 4.3; *p* < .05). Moreover, the mean office SBP change was −7.62 ± 9.32 mmHg, and the mean DBP change was −4.66 ± 6.03 (*p* < .05). Among the three subjects with abnormal homocysteine levels in the test group, homocysteine levels decreased by 6.23 ± 9.15 μmol/L after treatment. No differences were observed between the two groups in any other indicators. After 4 weeks of treatment, there were no significant differences between the groups in terms of safety indicators (*p* > .05). No abnormal vital signs (except BP) or severe liver or renal function impairment were observed during the treatment periods; in addition, adverse events and drug reactions were mild.

**Conclusion:** Treatment with Quanduzhong capsules reduced office SBP and DBP as well as DBP CV determined by 24-h ambulatory BP monitoring in patients with grade 1 hypertension at low-to-moderate risk.

**Clinical Trial Registration:** https://www.chictr.org.cn/showproj.aspx?proj=32531, identifier ChiCTR1900021699.

## 1 Introduction

Hypertension is a complex and multifactorial disease. Stroke and cardiovascular and kidney diseases all have a pathogenesis that is closely related to high blood pressure (BP) (Ettehad et al., 2016; Joint Committee for Guideline Revision, 2018). Hypertension, as a pandemic, has become the most critical and significant public health problem. Moreover, approximately one billion people worldwide suffer from hypertension, and less than a fifth have it under control, according to the World Health Organization (Hypertension, 2021).

Hypertension is defined as office systolic BP (SBP) values ≥140 mmHg and/or diastolic BP (DBP) values ≥90 mmHg (Williams et al., 2018). Currently, antihypertension medication still serves as a primary option for treating hypertension. It is still debated whether low-to-moderate risk grade 1 hypertension (140–159/90–99 mmHg) patients should immediately start treatment with antihypertensive drugs because older trials of “mild hypertension” included patients with BP levels potentially higher than those who were defined as having grade 1 hypertension or high risk for hypertension (Morales Salinas et al., 2017).

Furthermore, the potential adverse reactions of antihypertensive medication, such as headache, dizziness, orthostatic hypotension and decreased sexual function, limit their clinical application, especially in patients with grade 1 hypertension (Xiong et al., 2013a). Thus, looking for alternative therapies to control BP is necessary in the early stage of hypertension. Traditional Chinese medicine (TCM) has attracted much attention in recent years. Integrating TCM with Western medicine significantly improves the therapeutic effectiveness of hypertension treatment compared to Western medicine alone (Mohammed et al., 2023). Songling Xuemaikang capsules are beneficial for essential hypertension because they can lower BP and relieve hypertensive symptoms and they are well tolerated (Meng et al., 2022). A meta-analysis showed that Zhen Gan Xi Feng decoction, a traditional Chinese herbal formula, is effective in improving BP and hypertension-related symptoms (Xiong et al., 2013b). Recently, a Chinese herbal formula consisting of gastrodia-uncaria granules was shown to be efficacious for patients with masked hypertension in a randomized, placebo-controlled trial (Zhang et al., 2020).

The Quanduzhong capsules contain Duzhong [DZ (*Eucommia ulmoides* Oliv.)], which is a marketed formula well known for its antihypertensive effect. As shown by Zhang et al. (Zhang and Wei, 2018), the clinical curative effect of Quanduzhong capsules on renal hypertension is remarkable, as it can improve renal function and regulate BP circadian rhythm. Recently, Zhang et al. (Zhang et al., 2022a) observed the clinical effect of Quanduzhong capsules on new-onset mild hypertension. The patients in this study were treated for 6 months; 48 patients in the test group were given enalapril maleate tablets and Quanduzhong capsules, and 29 patients in the control group were given enalapril maleate tablets alone. The total effective rate of the test group was 97.92%, and the total effective rate of the control group was 85.42% (Zhang et al., 2022a). The clinical curative rate in the test group was higher (*p* = .027). A randomized controlled trial conducted by Jiang et al. (Jiang et al., 2022) confirmed that Quanduzhong capsules can lower office BP in patients with mild hypertension and kidney deficiency syndrome. The test group was given Quanduzhong capsules, and the control group received sham Quanduzhong capsules (Jiang et al., 2022). Compared with the BP values in the control group, the SBP and DBP in the test group decreased after 12 weeks of treatment (*p* < .05) (Jiang et al., 2022).

However, prior studies examining the efficacy of Quanduzhong capsules in treating hypertension were single-center studies or were not placebo-controlled. In addition, risk factors for hypertension were not taken into consideration. Therefore, we conducted a multicenter, randomized, double-blind, placebo-controlled clinical trial to assess the efficacy and safety of Quanduzhong capsules in grade 1 hypertension with low-to-moderate risk.

## 2 Materials and methods

### 2.1 Study design

This multicenter, randomized, placebo-controlled trial was conducted at three hospitals across mainland China, including Xiyuan Hospital, The First Affiliated Hospital of Guangxi University of Chinese Medicine, and Jiangxi Provincial People’s Hospital. Twenty patients were enrolled in each hospital. Our study, which was registered in the Chinese Clinical Trial Registry (ChiCTR1900021699), complies with the principles of the Declaration of Helsinki and Good Clinical Practice guidelines, and the ethics committee at each participating hospital approved the protocol. An independent data and safety monitoring committee blinded to treatment allocation oversaw the trial. The study was reported according to the CONSORT guidelines (Schulz et al., 2010).

### 2.2 Participant inclusion and exclusion criteria

Male and female patients between the ages of 30 and 75 years who were diagnosed with essential low-to-moderate risk grade 1 hypertension (140–159/90–99 mmHg) were eligible for participation. The subjects volunteered to participate in this trial and signed the informed consent form. Patients were excluded if they 1) were known or suspected to be allergic to the test drug and its components; 2) had malignant hypertension, hypertensive emergencies, hypertensive crisis, or hypertensive encephalopathy; 3) had secondary hypertension, including but not limited to the following diseases: unilateral or double renal artery, polycystic kidney disease, hyperaldosteronism, aortic coarctation, Cushing’s syndrome, or pheochromocytoma; 4) had the following comorbid diseases: acute myocardial infarction within 6 months, cerebrovascular accident, transient ischemic attack, large aneurysm or dissection aneurysm, unstable angina, grade II-IV (NYHA classification) history of heart failure, grade II or above atrioventricular block, sick sinus syndrome, bradycardia (heart rate <50 beats/min), or malignant or potentially malignant arrhythmias such as atrial fibrillation; 5) had serious liver, kidney or blood system diseases or malignant tumors; 6) had gastrointestinal lesions or gastrointestinal surgery such as gastrointestinal resection, active gastrointestinal inflammation, ulcers or gastrointestinal bleeding in the past 1 year; 7) had a reversed circadian rhythm or irregular sleep pattern; 8) were pregnant, were lactating, or had future plans for child bearing in 6 months; 9) had a history of alcohol or drug abuse 10) had neurological or mental disorders and could not or were unwilling to cooperate 11) were unable to participate in this research; and 12) had participated in other clinical trials 3 months prior.

### 2.3 Plant and drug preparation

Quanduzhong capsules are a China Food and Drug Administration-approved drug that contain an extraction of *Eucommia ulmoides* Oliv. [Eucommiaceae; Eucommiae cortex]. Each capsule of 0.48 g is equivalent to 2.5 g of crude drug. The crude drug was obtained from DZ Good Agricultural Practice (GAP) bases established by Jiangxi Puzheng Pharmaceutical Co., Ltd., in Jinggangshan and Jishui in Jiangxi Province, China. Quanduzhong capsules for the present clinical trial were prepared by Jiangxi Puzheng Pharmaceutical Co., Ltd., Jiangxi Province, China (production batch number: 180401; inspection order number: CP054180401).

With the cork removed, a total of 2500 g DZ was crushed into fine powder, 250 g of which was saved for later use, and the remainder was crushed, heated, refluxed with 85% ethanol for 2 h, and filtered, and the ethanol was recovered. The liquid medicine was stored for later use. Then, medicinal dregs were decocted twice with water, each time for an hour, and the decoctions were then combined and filtered. Once the filtrate had been combined with the abovementioned liquid medicine, it was concentrated into a paste with a relative density of 1.30 g/mL (80°C) by decompression; the paste was mixed with the 250 g fine powder mentioned above and soluble starch, then dried, crushed, sieved, and packed into 1000 capsules. Therefore, extractions were performed using ethanol extraction first, followed by water extraction. The Quanduzhong capsules contained a crude drug powder, which was improved by innovative micropowder technology to ensure uniformity and stability of the extraction and to ensure that the active ingredients were dispersed properly when taken, thus maximizing efficacy. The unfilled gelatin capsules used in this batch of products were made by Suzhou Capsule Co., Ltd. (production batch number: 12863878; material number: YF033-18012501).

The appearance of the sham capsules were indistinguishable in color, shape, size, packaging, smell, and taste from the Quanduzhong capsules, which were supplied by Jiangxi Puzheng Pharmaceutical Co., Ltd., China (production batch number: 180401; material number: CP054180701).

### 2.4 LC-MS/MS analysis of quanduzhong capsules

For LC-MS/MS analysis, 0.3 g Quanduzhong capsule powder was added to 25 mL deionized water and ultrasonically dissolved for 30 min. Then, 1 ml of the solution was taken, diluted 100 times, separated by high-speed centrifugation, and filtered through a 0.22 μm filter membrane.

The identification experiment was performed using ultra-performance liquid chromatography-quadrupole-time of flight (UPLC-Q-TOF) mass spectrometry (MS) [AB SCIEX Triple TOF 5600 + mass spectrometer system (AB SCIEX, Foster City, CA, United States)]. A C_18_ column (100 × 2.1 mm, 1.8 μm) was used with a flow rate of 0.25 ml/min. The mobile phases used were A) acetonitrile and B) 0.1% aqueous formic acid under the following gradient elution: 10% A from 0.1 to 2 min; 10%–25% A from 2 to 5 min; 25%–40% A from 5 to 15 min; 40%–90% A from 15 to 23 min; 90% A from 23 to 27 min. The electrospray ionization (ESI) source was used in negative ion mode. For the compounds of interest, a scan range of m/z 120–1500 was chosen. Other conditions were as follows: atomization temperature, 600°C; spray voltage, −4500 V; declustering potential, −80 V. MS data were collected in TOF-MS-IDA-MS/MS mode.

### 2.5 Randomization, intervention and masking

In each center, 20 patients were randomly assigned using a block randomization method in a 1:1 ratio to the test group or control group. After checking for eligibility during screening, subjects were allocated numbers in chronologic order. First, each participant took three sham Quanduzhong capsules twice daily for 1 week. After the sham lead-in period, all patients who met the entry criteria were allowed to enter the next 4 week test period. During the 4 week test period, the test group took three Quanduzhong capsules twice daily, and the control group continued to take sham Quanduzhong capsules. In addition, all randomized patients adopted similar lifestyle interventions throughout the trials. A two-level blinding method was set up, with the first level being by group (group A and group B) according to case number and the second level being by treatment (Quanduzhong capsules and sham). All investigators, data collectors, and patients were masked to the treatment allocation.

### 2.6 Outcomes

The primary outcome measures were 24-h mean SBP and DBP determined *via* 24-h ambulatory blood pressure monitoring (ABPM) and office SBP and DBP. The secondary efficacy measures were mean arterial pressure, mean pulse, daytime mean SBP and DBP, nocturnal mean SBP and DBP, SBP and DBP load, area under the BP curve, morning peak BP, early morning SBP and DBP, smoothness index of SBP and DBP, 24 h BP mean CV, percentage of patients with circadian restoration in ABPM, home BP, quality of life evaluated by WHO Quality of Life-BREF (WHOQOL-BREF) questionnaire, grading and quantitative evaluation of hypertension symptoms, as well as values of plasmatic renin activity (PRA), angiotensin II (Ang II), aldosterone (ALD), β_2_-microglobulin (β_2_m) and homocysteine (Hcy).

### 2.7 Safety measures

The clinical laboratory tests included routine blood and urine parameters, as well as liver and kidney function examinations. The measured vital signs included pulse, body temperature, and respiratory rate. In addition, adverse events (AEs) were recorded at any time of occurrence during the trial. An AE was categorized as related (definitely, probably, or possibly) or not related (unlikely or not related) to the study drug (Ren et al., 2012; Belknap et al., 2017). An adverse drug reaction (ADR) was defined as an AE that was considered related to the study drug.

### 2.8 Statistical analysis

The full analysis set (FAS), per protocol set (PPS), and safety set (SS) were included for analysis by SAS software, version 9.4 (SAS Institute). All hypothesis tests were two-sided, with *α* = 0.05; thus, results where *p* < .05 were considered statistically significant. All results are presented with a 95% confidence interval (95% CI). Measurement data are expressed as the mean, standard deviation (SD), median, Q1 (25th percentile), Q3 (75th percentile), and maximum and minimum values. Descriptive statistics were calculated as frequencies to express count data; inferential statistics and the corresponding *p* values were calculated. Depending on the type of variable and its distribution in measurement data, the Kruskal–Wallis (K-W) test and analysis of variance (ANOVA) were used to compare characteristics between groups; comparisons of exams among groups were performed by Fisher’s exact test or the chi-square test for descriptive statistics; the Cochran–Mantel–Haenszel (CMH) test was used for grade data. Primary efficacy measures were analyzed by ANCOVA with the baseline as a covariate. The chi-square test was used to compare the incidence of adverse events and reactions between the two groups.

## 3 Results

### 3.1 Identification of the main compounds in quanduzhong capsules

**FIGURE 1:**
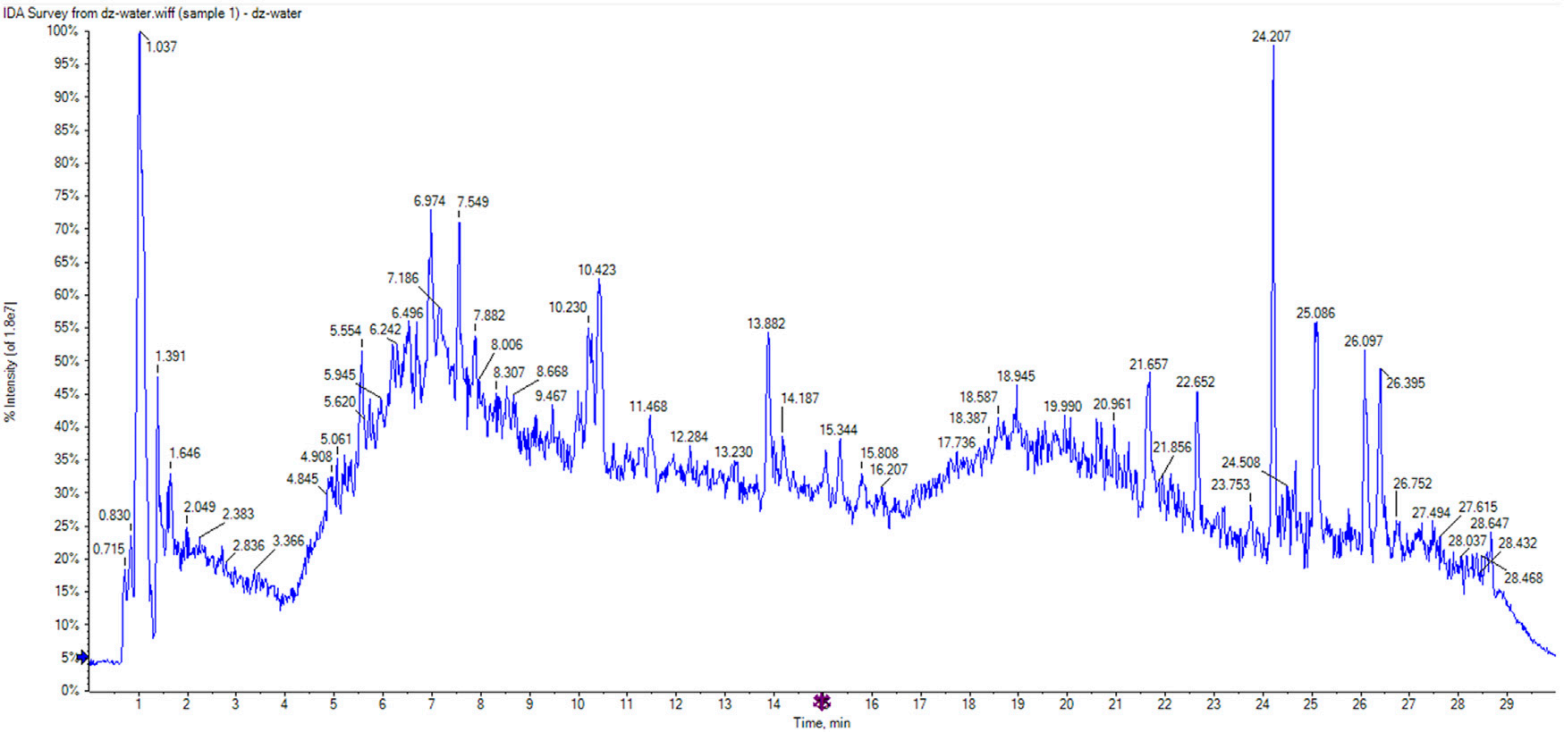
TIC of Quanduzhong capsules.

**FIGURE 2:**
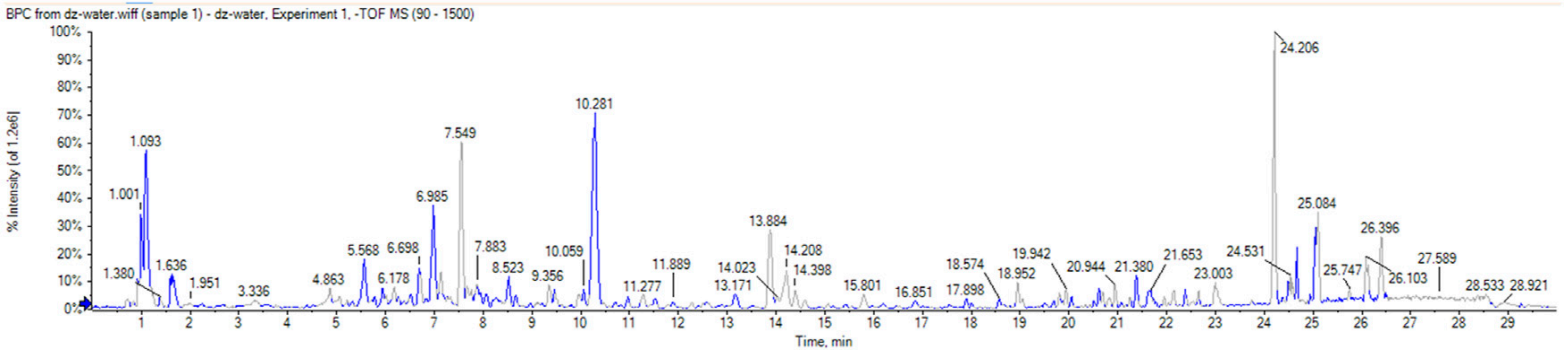
BPC of Quanduzhong capsules.

A total of 51 chromatographic peaks of Quanduzhong capsules were identified by LC‒MS/MS. The total ion chromatogram (TIC) and base peak chromatogram (BPC) are shown in Figures 1, 2. Supplementary Table S1 shows the results of the chemical components in Quanduzhong capsules.

### 3.2 Demographic and baseline characteristics

**FIGURE 3:**
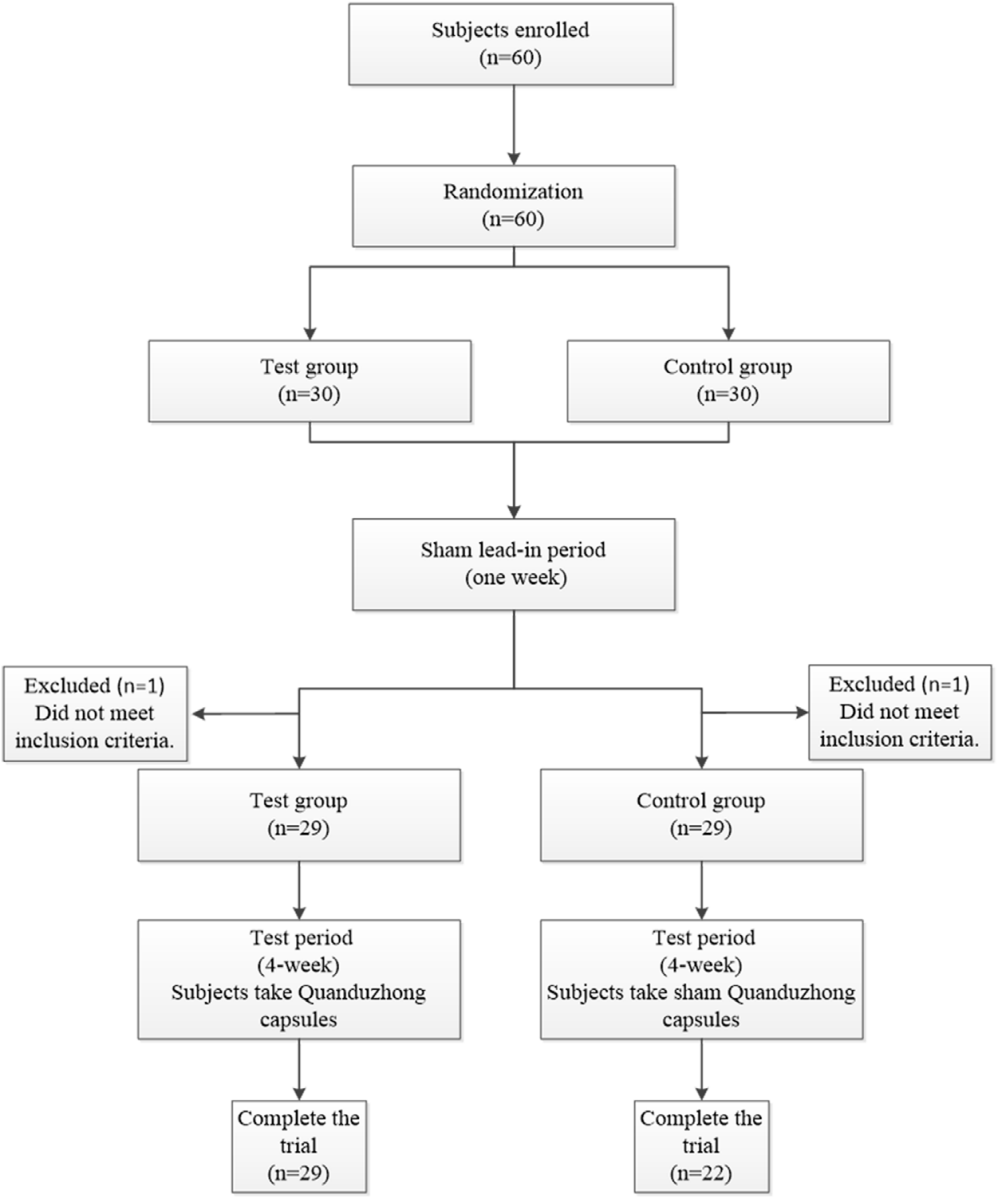
Flow chart of the study.

A total of 60 patients were enrolled at 3 centers (Xiyuan Hospital, The First Affiliated Hospital of Guangxi University of Chinese Medicine, and Jiangxi Provincial People’s Hospital) from June 2018 to June 2020. The flow chart of this study is presented in Figure 3. Sixty patients were randomized to the test group (n = 30) or the control group (n = 30). Over the course of this study, there was a 1 week sham lead-in period and a 4 week test period. After the sham lead-in period, two patients were excluded who did not meet the inclusion criteria. Overall, 51 (85%) patients completed the study, and 9 (15%) patients did not. Specifically, two patients from Jiangxi Provincial People’s Hospital and one patient from The First Affiliated Hospital of Guangxi University of Chinese Medicine discontinued treatment because of unsatisfactory therapeutic effects; three patients from The First Affiliated Hospital of Guangxi University of Chinese Medicine were lost to follow-up because of the COVID-19 epidemic; two patients from Xiyuan Hospital were lost to follow-up for personal reasons; and one patient from The First Affiliated Hospital of Guangxi University of Chinese Medicine was excluded because of incorrect inclusion. The FAS included 58 (97%) patients, with 29 from the test group and 29 from the control group. The PPS included 51 (85%) patients, with 29 from the test group and 22 from the control group.

Baseline characteristics were comparable across the test and control groups and are presented in Table 1. Of all 58 subjects, 19 were women and 39 were men. The mean age of the subjects was 52.78 years. The mean height was 165.30 cm. The mean weight was 69.36 kg. Fifty-four patients were of Chinese Han ethnicity. In addition, 30 subjects were at low risk and 28 were at moderate risk. Two subjects had a history of drug or other allergies. Twenty-three patients had a history of other diseases. Forty-five patients received no treatment with medicine or non-pharmaceutical therapies within the last 3 months. In addition, eight male patients were smokers (1 in the test group and seven in the control group). There were no significant differences in baseline characteristics between the test and control groups (*p* > .05), except for smoking (*p* = .02).

**TABLE 1:** Baseline characteristics of the study subjects.

Characteristics	Test group	Control group	*p*
Age—yrs	53.76 ± 11.29	51.79 ± 9.49	.48
Sex			.78
Male	20	19	
Female	9	10	
Ethnicity			.61
Han	26	28	
Other ethnic groups	3	1	
Risk			.29
Low risk	17	13	
Moderate risk	12	16	
High risk	0	0	
Height (cm)	165.07 ± 6.24	165.52 ± 8.58	.82
Weight (kg)	67.81 ± 10.17	70.9 ± 13.12	.32
History of drug or other allergies			.49
Yes	0	2	
No	29	27	
History of other diseases			.42
Yes	10	13	
No	19	16	
Treatment with medicine or non-pharmaceutical therapies within 3 months			.34
Yes	8	5	
No	21	24	
Smoking or not			.02
Currently smoking	1	7	
1–9 cigarettes per day	0	4	
≥10 cigarettes per day	1	3	
Quit smoking	0	1	
Never smoked	28	21	

### 3.3 Efficacy

#### 3.3.1 ABPM index analysis

**FIGURE 4:**
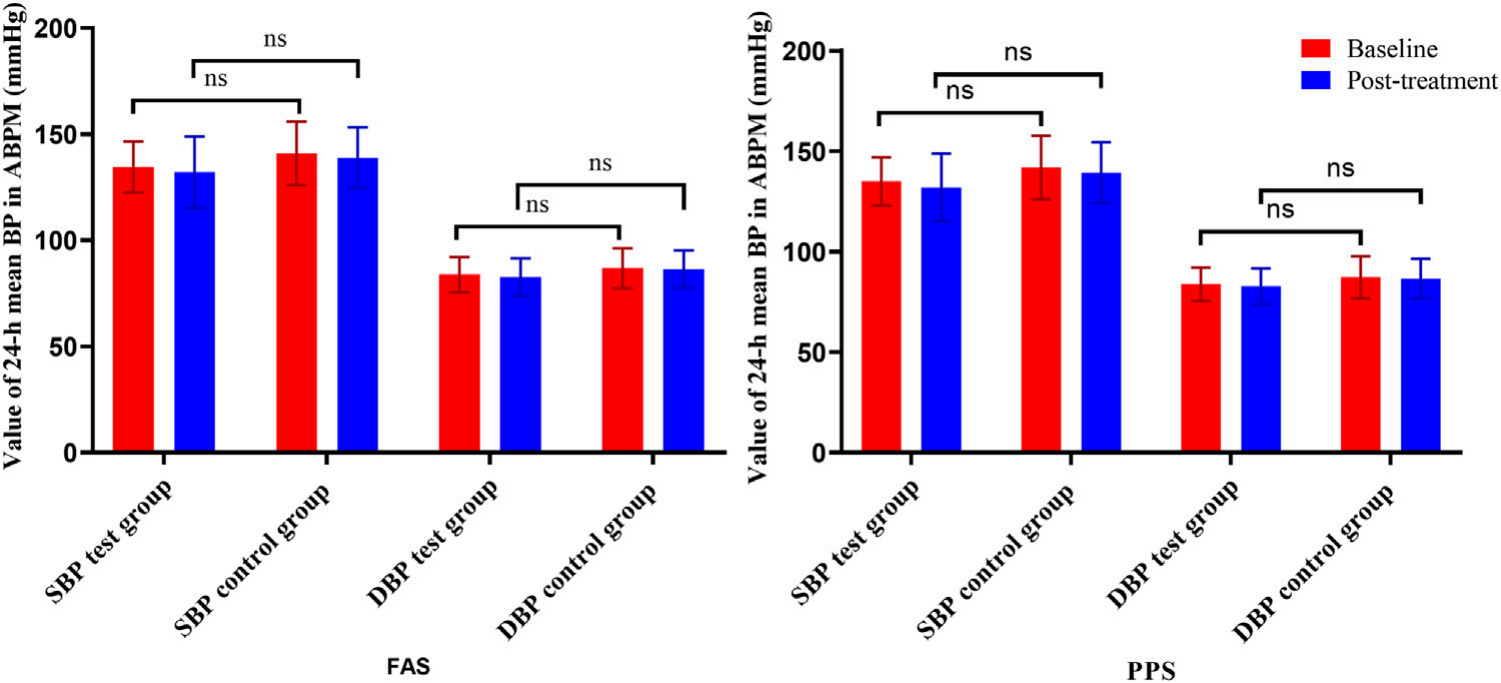
Changes in the 24-h mean BP from ABPM (left is FAS; right is PPS). *Note: ns = non-significant.*

As the primary outcome, changes in the 24-h mean SBP and DBP are presented in Table 2 and Figure 4; in the test group, the 24-h mean SBP decreased by 2.52 ± 12.09 mmHg, and the 24-h mean DBP decreased by 1.17 ± 6.65 mmHg; in the control group, the 24-h mean SBP decreased by 1.93 ± 9.63 mmHg, and the 24-h mean DBP decreased by 0.41 ± 7.26 mmHg. The differences in SBP and DBP between the test and control groups were not significant (*p* > .05).

**TABLE 2:** Changes in 24-h ABPM values.

	FAS difference value			PPS difference value		
	Test group	Control group	*p*	Test group	Control group	*p*
24-h MSBP	−2.52 ± 12.09	−1.93 ± 9.63	.58	−2.52 ± 12.09	−2.55 ± 11.04	.71
24-h MDBP	−1.17 ± 6.65	−0.41 ± 7.26	.34	−1.17 ± 6.65	−0.55 ± 8.38	.41
24-h MAP	−1.69 ± 8.38	−1 ± 7.97	.37	−1.69 ± 8.38	−1.32 ± 9.18	.48
24-h MP	1.89 ± 7	2.04 ± 5.85	.90	1.96 ± 7.13	2.75 ± 6.7	.70
Daytime MSBP	−2 ± 17.28	−1.93 ± 10.62	.39	−2 ± 17.28	−2.55 ± 12.2	.45
Daytime MDBP	−1.76 ± 6.84	−0.24 ± 7.83	.16	−1.76 ± 6.84	−0.32 ± 9.04	.22
Daytime MAP	−2.31 ± 8.72	−0.66 ± 8.63	.17	−2.31 ± 8.72	−0.86 ± 9.96	.23
Daytime MP	1.41 ± 7.44	1.79 ± 6.06	.72	1.46 ± 7.59	2.5 ± 7.09	.47
N MSBP	1.68 ± 12.91	−0.93 ± 14.47	.79	1.74 ± 13.15	−1.23 ± 16.7	.79
N MDBP	1.64 ± 8.49	−0.86 ± 11	.63	1.7 ± 8.64	−1.14 ± 12.69	.78
N MAP	1.36 ± 9.72	−0.9 ± 11.97	.83	1.41 ± 9.9	−1.18 ± 13.81	.88
N MP	2.5 ± 6.52	3.11 ± 8.02	.77	2.71 ± 6.75	4.35 ± 9.26	.51
SBP Load	−4.76 ± 16.52	−1.99 ± 18.5	.27	−4.96 ± 16.84	−2.79 ± 22	.36
DBP Load	−1.88 ± 16.77	2.25 ± 22.48	.27	−1.96 ± 17.12	3.16 ± 26.74	.33
SBP AUC	−46.48 ± 481.94	−68.28 ± 288.28	.90	−46.48 ± 481.94	−90 ± 329.78	.99
DBP AUC	−22.66 ± 296.52	−30.83 ± 196.16	.73	−22.66 ± 296.52	−40.64 ± 225.58	.77
Early morning SBP	−3.19 ± 15.67	−1.71 ± 10.9	.26	−3.54 ± 19.41	−2.94 ± 13.68	.29
Early morning DBP	−2.11 ± 12.95	−0.63 ± 7.43	.18	−2.68 ± 14.4	−0.02 ± 8.98	.03
Morning peak BP	−5.85 ± 14.67	−1.67 ± 15.55	.40	−6.31 ± 16.53	−3.83 ± 18.57	.32
Smoothness index in SBP	−0.02 ± 0.13	−0.03 ± 0.08	.82	−0.02 ± 0.13	−0.04 ± 0.09	.89
Smoothness index in DBP	0.01 ± 0.12	−0.01 ± 0.08	.82	0.01 ± 0.12	−0.02 ± 0.09	.87

FAS = the full analysis set; PPS = the per protocol set; MSBP = mean systolic blood pressure; MDBP = mean diastolic blood pressure; MAP = mean arterial pressure; MP = mean pulse; N = nocturnal; AUC = area under the BP, curve.

**FIGURE 5:**
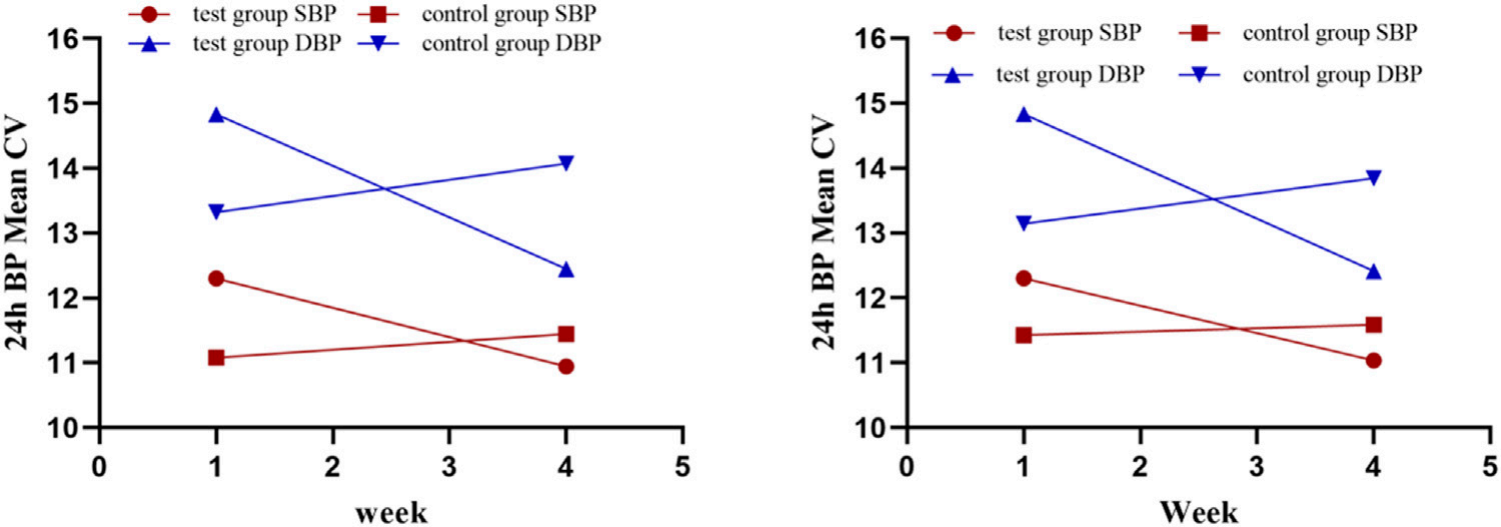
24-h BP mean CV (left is FAS; right is PPS).

Various other indices of 24-h ABPM presented as secondary outcomes are shown in Tables 2, 3 and Figure 5. For FAS (results were consistent with the PPS), at baseline in the test and control groups, the CV of DBP by ABPM was 14.83 ± 5.04 and 13.32 ± 3.31, respectively. After 4 weeks, the CV values were 12.45 ± 2.93 and 14.07 ± 2.8; the difference values were −2.49 ± 4.32 and 0.76 ± 4.3. The CV of DBP after the test period decreased in the test group and increased in the control group, and the differences between the two groups were significant (*p* = .01). For the CV of SBP, the difference values between baseline and 4 weeks later were −1.65 ± 3.41 (test group) and 0.37 ± 2.83 (control group), but the differences were not significant (*p* = .07). The changes in CV are shown in Figure 5. In addition, as shown in Table 2, other 24-h ABPM values, such as the mean BP, mean arterial pressure, mean pulse, daytime BP, nocturnal BP, early morning and morning peak BP, area under the BP curve and smoothness index, were not found to be significantly different between the two groups. The percentage of patients with circadian restoration is listed in Table 3. The test group was 9.09%/9.09% (FAS/PPS), and the control group was 10%/25% (FAS/PPS); there were no significant differences (*p* > .05).

**TABLE 3:** Percentage of patients with circadian restoration.

	FAS/PPS		
	Test group	Control group	*p*
Non-dipper hypertension in baseline	11/11	10/4	
Dipper hypertension after treatment (%)	1 (9.09)/1 (9.09)	1 (10)/1 (25)	1/.48

#### 3.3.2 Office BP

**FIGURE 6:**
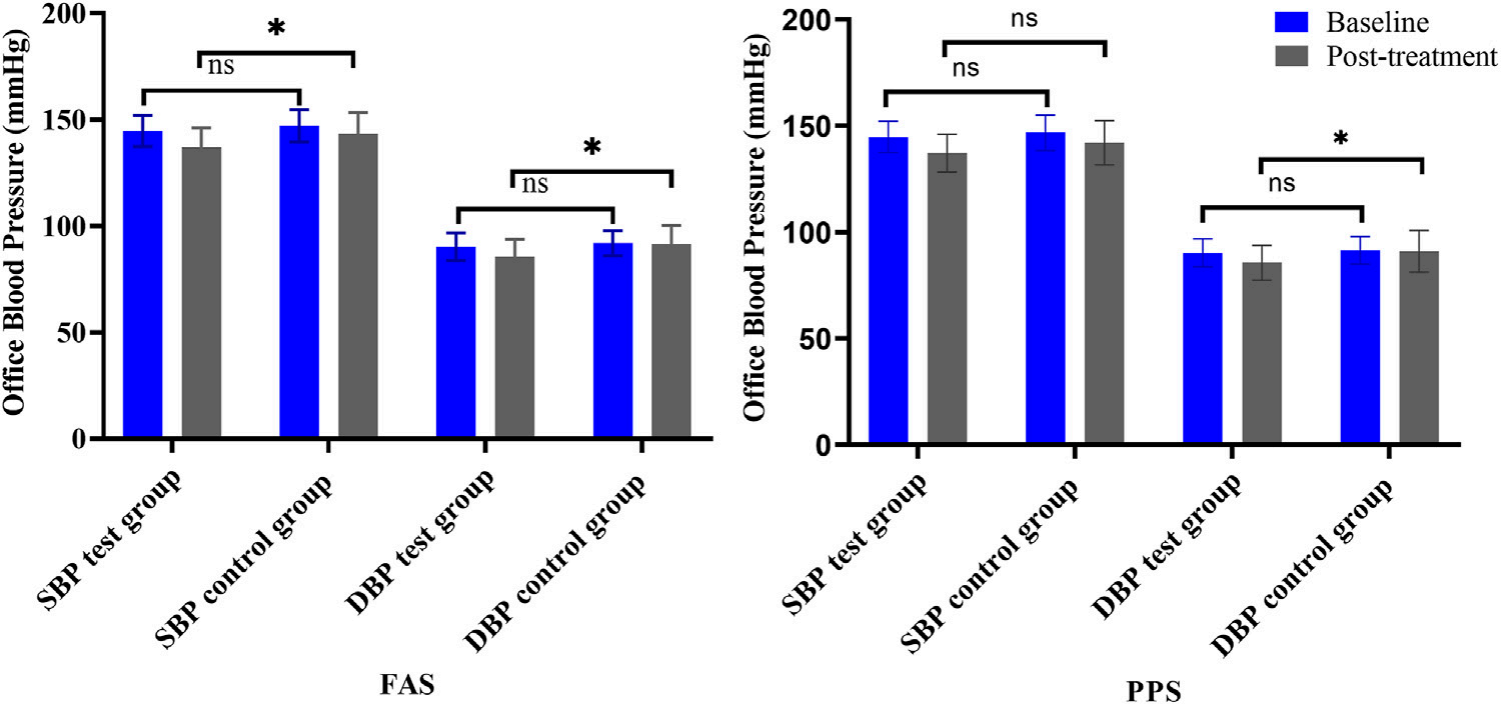
Changes in office BP (left is FAS; right is PPS). Note: ns = non-significant. *p < .05 versus *posttreatment.*

Office SBP and DBP were other primary outcomes. At baseline, there were no statistically significant differences between the two groups. After 4 weeks of treatment, the SBP and DBP in both groups were decreased. In the FAS, SBP decreased 7.62 ± 9.32 mmHg in the test group and decreased 3.55 ± 7.55 mmHg in the control group, and DBP decreased 4.66 ± 6.03 mmHg and 0.38 ± 6.65 mmHg, respectively. The difference in SBP and DBP between the two groups was significant (*p* < .05). In PPS, the difference in DBP between the two groups was significant (*p* = .03), but no significant difference was found in SBP (*p* = .13) (Table 4; Figure 6).

**TABLE 4:** Changes in office BP (FAS, PPS).

	FAS			PPS		
	Test group	Control group	*p*	Test group	Control group	*p*
MSBP in baseline	144.69 ± 7.31	147.1 ± 7.55	.22	144.69 ± 7.31	146.64 ± 8.26	.38
MDBP in baseline	90.31 ± 6.52	91.9 ± 5.95	.34	90.31 ± 6.52	91.5 ± 6.49	.52
MSBP in 4 weeks	137.07 ± 8.92	143.55 ± 9.73	.01	137.07 ± 8.92	141.96 ± 10.41	.08
MDBP in 4 weeks	85.66 ± 8.2	91.52 ± 8.75	.01	85.66 ± 8.2	91 ± 9.83	.04
Difference in SBP	−7.62 ± 9.32	−3.55 ± 7.55	.03	−7.62 ± 9.32	−4.68 ± 8.4	.13
Difference in DBP	−4.66 ± 6.03	−0.38 ± 6.65	.01	−4.66 ± 6.03	−0.5 ± 7.67	.03

#### 3.3.3 Home BP and other outcomes

After 4 weeks of treatment, for home BP, the normotensive test group comprised 18 (62.07%) subjects in the FAS and 18 (62.07%) subjects in the PPS, while the normotensive control group comprised 11 (37.93%) subjects in the FAS and 7 (31.82%) subjects in the PPS. In addition, the number (percentage) of subjects in the test group who experienced a decrease in home BP was 19 (65.52%) in the FAS and 19 (65.52%) in the PPS, while in the control group, the number (percentage) of subjects was 15 (51.72%) in the FAS and 11 (50%) in the PPS, but there were no significant differences between the two groups (*p* > .05). Changes observed in home BP after treatment are shown in Table 5, and there were no significant differences.

**TABLE 5:** Changes in home BP from baseline to 4 weeks (FAS, PPS).

	FAS difference value			PPS difference value		
	Test group	Control group	*p*	Test group	Control group	*p*
SBP	−2.68 ± 9.8	−0.21 ± 11.35	.19	−2.78 ± 9.97	−0.24 ± 12.13	.25
DBP	−3.17 ± 11.8	−1.24 ± 9.08	.15	−3.28 ± 12	−1.41 ± 9.69	.22

Regarding the quality-of-life evaluation, which was performed using the WHOQOL-BREF questionnaire, and the grading and quantitative evaluation of hypertension symptoms, no significant differences were found between the two groups, either at baseline or after 4 weeks of treatment.

Regarding laboratory indicators, a few participants had abnormal baseline results, so we decided to describe the results and not to perform hypothesis testing. In the test group, there were no patients with baseline abnormal PRA, Ang II, or ALD. In the control group, one patient had abnormal PRA with a value of 42.78 pg/ml; after 4 weeks of treatment, the value was 20.99 pg/ml. One patient had abnormal Ang II, and the baseline and 4 week values were 923.97 pg/ml and 1012.28 pg/ml, respectively. There was one patient who was found to have abnormal ALD in the control group; the baseline value was 310.81 pg/ml with no change after 4 weeks. The results of β_2_m and Hcy are shown in Table 6.

**TABLE 6:** Changes in β_2_m and Hcy.

	β_2_m (mg/L)		Hcy (μmol/L)	
	Test group	Control group	Test group	Control group
N	4	3	3	8
Mean difference	−0.07 ± 0.18	−0.15 ± 1.39	−6.23 ± 9.15	−0.87 ± 2.03

## 4 Safety

No abnormal vital signs (except BP) or severe liver or renal function impairment were observed during the treatment periods. As shown in Supplementary Table S2, in the SS analysis (n = 59), 22 (37.29%) patients experienced 23 AEs, and 15 ADRs were reported in 14 (23.73%) patients. In addition, AEs were confirmed in 12 (40%) patients from the test group and 10 (34.48%) from the control group; 8 (26.67%) patients in the test group and 6 (20.69%) patients in the control group experienced ADRs. AEs and ADRs were not significantly different between groups (AE, *p* = .66; ADR, *p* = .59) (Supplementary Table S3). In our study, both AEs and ADRs were mild, without leading to unblinding. No patients withdrew from the trial. Detailed information is listed in Supplementary Tables S4, S5.

## 5 Discussion

Our results demonstrated that treatment with Quanduzhong capsules can result in a greater reduction in office BP and in the DBP CV of 24-h ABPM compared with placebo. There is general agreement that patients with grade 2 or 3 hypertension should be treated with antihypertensive drugs and lifestyle interventions. In addition, guidelines consistently recommend that patients with grade 1 hypertension who are at high cardiovascular risk or who have hypertension-mediated organ damage should be treated with BP-lowering medication. It has been less consistent regarding whether BP-lowering drugs should be prescribed to patients with grade 1 hypertension and low-to-moderate risk; that is, the benefit of antihypertensive medication in individuals with grade 1 hypertension with low-to-moderate risk is controversial, so there is room for alternative therapies (Zanchetti, 2015). TCM plays a significant role in this field. A recent meta-analysis reported that long-term variability in BP is associated with cardiovascular and mortality outcomes (Stevens et al., 2016). Thus, decreasing BP variability is essential in the treatment of hypertension. The efficacy of Quanduzhong capsules in reducing the CV of DBP was demonstrated in our study. Many Chinese herbal formulae have also been found to reduce the CV of BP; these include Qingxuanjiangya decoction, Niuhuangjiangya pills, and Banxia Baizhu Tianma decoction, which are used for treating liver fire/liver-yang hyperactivity pattern and phlegm-fluid retention pattern in hypertensive patients (Xiong et al., 2013a).

For Hcy, the baseline values of three patients were abnormal in the test and control groups. After 4 weeks, the Hcy level in the test group was significantly lower than that in the control group. Thus, treatment with Quanduzhong capsules may be associated with a decrease in Hcy levels. However, we cannot draw firm conclusions given our small sample size. The elevation of plasma Hcy is identified as an independent risk factor for cardiovascular disease and is associated with an increased risk of ischemic stroke (Ganguly and Alam, 2015; Koller et al., 2018; Yuan et al., 2021). Thus, this finding is worthy of further investigation.

“Syndrome” or “pattern” refers to a pathological state that manifests itself as a corresponding set of signs and symptoms, representative of a specific stage in the progression of the disease in TCM. There are various Chinese medicine patterns for hypertension, each with its own specific symptoms and constitutions, with dizziness or headache being the primary complaint. According to the “*Expert Consensus on Diagnosis and Treatment of Hypertension with Traditional Chinese Medicine*”, the patterns of hypertension include kidney essence insufficiency, kidney yang deficiency, yin and yang deficiency, liver yang ascendant hyperactivity, liver yang and yin deficiency, etc. (Society of Cardiovascular Diseases and China Association of Chinese Medicine, 2019). The pathogenesis of hypertension is largely attributed to kidney deficiency, and tonifying the kidney has become a new strategy for reducing BP (Society of Cardiovascular Diseases and China Association of Chinese Medicine, 2019). As a classic pattern in TCM, kidney deficiency is characterized by feeling vexed, difficulty falling asleep, ringing in the ears, a cold feeling in the back, aversion to wind, and soreness in the lower back and knees. In East Asia, particularly in China, Korea, and Japan, DZ is commonly used as an herbal kidney-tonifying remedy to treat hypertension, either alone or in combination with other medications.

The Quanduzhong capsules used in our study consist of an extract of DZ bark. DZ belongs to the monotypic genus Eucommia (Cronquist, 1981), which has been used as a traditional medical therapy for approximately 2000 years. As an upper grade drug, DZ was first recorded in the Shen Nong Ben Cao Jing (Sheng Nong’s herbal classic). The bark, leaves, seeds, and even male flowers of this plant are commonly used as medicine. DZ bark was documented in the Pharmacopoeia Committee of the People’s Republic of China 2020 for its effects of tonifying the liver and kidneys, strengthening muscles and bones, and calming fetuses; similar to DZ bark, DZ leaves nourish the liver and kidneys, strengthen the tendons and bones and are often employed as an alternative medicine. The medicinal properties of DZ include antihypertensive, antiinflammatory, antihyperlipidemic, antidiabetic, antioxidant, and antitumor properties (He et al., 2014; Shao et al., 2015; Zhang et al., 2022b).

The antihypertensive efficacy of DZ has been known for over 40 years, and evidence for the efficacy of DZ against hypertension was identified in modern pharmacological studies. (+)-Pinoresinol di-β-d-glucoside is the main antihypertensive pharmacological active compound, which was reported as early as 1970 (Sih et al., 1976). Recent studies have found that lignans, flavonoids, and iridoids from DZ exert hypotensive effects, potentially due to inhibiting oxidative stress, improving endothelial dysfunction, regulating the autonomic nervous system, activating ion channels, and regulating the renin-angiotensin-aldosterone (RAAS) system (Sih et al., 1976; Li et al., 2013; He et al., 2014; Hosoo et al., 2015). Both the bark and leaves of DZ exhibit these biological activities.

Several animal models and patients with hypertension are reported to have gut dysbiosis, characterized by reduced diversity and an abnormal structure (Sun et al., 2019). In a recent study by Yan D et al. (Yan et al., 2022), a high-salt diet and N (omega)-nitro-L-arginine methyl ester-induced hypertensive mice were treated with bark extract, which improved blood pressure, ameliorated kidney injury, decreased serum IL-6 and IL-17α as well as renal IL-17α, and restored gut microbiota diversity and composition. The potential gut bacteria involved in the antihypertensive action of DZ was suggested to be Parabacteroides strain XGB65, which exerted antihypertensive effects by lowering the levels of inflammatory cytokines such as renal IL-17α (Yan et al., 2022).

Antihypertensive effects have been shown for other parts of DZ. DZ leaves are effective against secondary hypertension, such as renal hypertension and salt-sensitive hypertension, as well as hypertension caused by thoracic aortic endothelial dysfunction, high-fat diets, and oxidized low-density lipoprotein by regulating the Ras homolog family member A/Rho-associated protein kinase signaling and NO/soluble guanylate cyclase/cyclic guanosine monophosphate signaling pathways (Li et al., 2022). In spontaneously hypertensive rats, the male flower extract of DZ exhibited antihypertensive effects by targeting the angiotensin-converting enzyme 2/angiotensin (1–7)/Mas signaling pathway (Ding et al., 2020). In addition, DZ can lower BP when combined with other herbs. For example, DZ and Jili (Tribulus terrestris L.) inhibit the onset and progression of hypertension by regulating ferroptosis in vascular neuron cells by acting on proteins associated with neuroactive ligand‒receptor interactions (Zhang et al., 2022b). Overall, DZ is considered a promising therapy for treating hypertension due to its extensive biological activities.

A previous study using approaches based on network robustness identified 21 possible active compounds in Quanduzhong capsules that may target 13 genes specifically expressed in glomeruli, including RAAS, lipid metabolism, immune response, and inflammatory response (Guo et al., 2019). In these biological processes, primary hypertension factors and kidney protection factors are implicated, demonstrating that Quanduzhong capsules may improve renal function and reduce hypertensive risk factors by inhibiting inflammation, reducing oxidative stress, and regulating metabolic homeostasis (Guo et al., 2019). There is, however, a need for further experimental verification of the molecular mechanisms involved in the BP-lowering effects of Quanduzhong capsules.

This study has some limitations that should be addressed in future studies. First, the sample was small, containing only 60 patients. Second, although recruited from three hospitals in China, all patients selected in this study were Chinese; thus, the broad representativeness is limited. Third, the 4-week study duration was short. A trial with a larger sample size and a longer course of treatment is needed in the future. Fourth, TCM patterns need to be incorporated into the inclusion criteria in future studies.

## 6 Conclusion

Treatment with Quanduzhong capsules was superior to placebo for reducing office SBP and DBP and the DBP CV of 24-h ABPM. As far as safety is concerned, Quanduzhong capsules did not result in any noticeable adverse effects compared to placebo.

## Data Availability

The original contributions presented in the study are included in the article/Supplementary Material, further inquiries can be directed to the corresponding author.

## Abbreviations

ALD, aldosterone; Ang II, angiotensin II; ADR, adverse drug reaction; AEs, adverse events; ANOVA, analysis of variance; BP, blood pressure; β2m, β2-microglobulin; CV, coefficient of variation; CI, confidence interval; CMH, Cochran–Mantel–Haenszel; DBP, diastolic blood pressure; DZ, Duzhong; FAS, full analysis set; GAP, Good Agricultural Practice; HPLC, high-performance liquid chromatography; Hcy, homocysteine; K-W, Kruskal–Wallis; PRA, plasmatic renin activity; PPS, per protocol set; RAAS, renin-angiotensin-aldosterone; SBP, systolic blood pressure; SS, safety set; SD, standard deviation; TCM, Traditional Chinese medicine; UK: unknown; WHOQOL-BREF, WHO Quality of Life-BREF; 24-h ABPM, 24-h ambulatory blood pressure monitoring.
