# Supplementary material for: Quanduzhong capsules for the treatment of grade 1 hypertension patients with low-to-moderate risk: A multicenter, randomized, double-blind, placebo-controlled clinical trial
Source: Front Pharmacol. 2023 Jan 10;13:1014410. doi: 10.3389/fphar.2022.1014410 (PMC9871381; doi:10.3389/fphar.2022.1014410)
Supplement: Supplementary file 1 [file DataSheet1.docx]

Supplementary Material

**Supplementary Table S1 Identification of chemical constituents in Quanduzhong capsule**

| S.No | tR (min) | [M-H]^-^ | Molecular formula | MS/MS fragments | Identification |
| --- | --- | --- | --- | --- | --- |
| 1 | 7.20 | 387.1440 | C_21_H_24_O_7_ | 357.0973,341.2002,313.1083,151.0407 | Medioresinol |
| 2 | 6.93 | 549.1967 | C_27_H_34_O_12_ | 387.1446,372.1213,181.0519 | Medioresinol 4’-O-β-D-glucopyranoside and its isomer |
| 3 | 7.20 | 549.1970 | C_27_H_34_O_12_ | 503.2497,387.1447,372.1211,151.0418 |  |
| 4 | 5.77 | 711.2499 | C_33_H_44_O_17_ | 549.1981,550.2030,387.1457 | (+)-Medioresinol Di-O-β-D-glucopyranoside |
| 5 | 6.98 | 357.1341 | C_20_H_22_O_6_ | 342.1097,151.0413,136.0179 | Pinoresinol and its isomer |
| 6 | 10.44 | 357.1335 | C_20_H_22_O_6_ | 327.1143,324.0899,311.1492,281.0730,151.0406,136.0178 |  |
| 7 | 11.72 | 357.1340 | C_20_H_22_O_6_ | 313.2380,311.0935,252.0813,201.1154 |  |
| 8 | 6.98 | 519.1859 | C_26_H_32_O_11_ | 357.1340,151.0412 | (+)-pinoresinol-β-D-glucoside |
| 9 | 5.57 | 681.2392 | C_32_H_42_O_16_ | 519.1855,357.1339 | Pinoresinol diglucoside |
| 10 | 7.89 | 373.1287 | C_20_H_22_O_7_ | 358.1055,343.1185,325.1083,313.1079,298.0851,108.0241 | 1-Hydroxypinoresinol and its isomer |
| 11 | 9.14 | 373.1286 | C_20_H_22_O_7_ | 355.1180,327.2135,285.2044,235.0626,201.1137,179.0717,164.0488,123.0489 |  |
| 12 | 10.42 | 373.1281 | C_20_H_22_O_7_ | 358.1049,327.2176,203.0719,151.0406 |  |
| 13 | 12.58 | 373.1287 | C_20_H_22_O_7_ | 358.1058,327.2170,167.0355,108.0233 |  |
| 14 | 6.15 | 535.1815 | C_26_H_32_O_12_ | 373.1294,343.1191,313.1086 | (+)-1-hydroxypinoresinol 4’’-O-β-D-glucopyranoside and its isomer |
| 15 | 8.65 | 535.1813 | C_26_H_32_O_12_ | 373.1295,358.1050 |  |
| 16 | 5.06 | 697.2343 | C_32_H_42_O_17_ | 651.2329,535.1781,373.1291,343.1186,211.0625 | 1-hydroxypinoresinol 4’,4’’-O-β-D-glucopyranoside |
| 17 | 7.13 | 417.1552 | C_22_H_26_O_8_ | 402.1317,387.1084,359.1154,181.0516,166.0279 | Syringaresinol |
| 18 | 5.94 | 579.2073 | C_28_H_36_O_13_ | 417.1551,402.1348,181.0518 | Syringaresinol-O-β-D-glucopyranoside and its isomer |
| 19 | 7.13 | 579.2074 | C_28_H_36_O_13_ | 417.1549,402.1319,181.0517 |  |
| 20 | 5.94 | 741.2605 | C_34_H_46_O_18_ | 579.2071,417.1553 | Liriodendrin and its isomer |
| 21 | 6.93 | 741.2606 | C_34_H_46_O_18_ | 519.1889,417.1534,357.1328,323.0966,221.0464,161.0456 |  |
| 22 | 5.71 | 375.1445 | C_20_H_24_O_7_ | 360.1207,327.1243,297.0744,257.0817,241.0514,189.0552,108.0231 | Olivil |
| 23 | 6.53 | 375.1446 | C_20_H_24_O_7_ | 360.1221,327.1248,195.0659,191.0717,179.0714,122.0387 | Cycloolivil |
| 24 | 2.53 | 345.1186 | C_15_H_22_O_9_ | 299.1119,161.0300,119.0340 | Aucubin |
| 25 | 13.63 | 507.1655 | C_21_H_32_O_14_ | 491.1377 | b-D-Glucopyranoside,(1S,4aR,5S,7aS)-1,4a,5,7a-tetrahydro-5-hydroxy-7-(hydroxymethyl)cyclopenta[c]pyran-1-yl |
| 26 | 7.55 | 187.0983 | C_9_H_16_O_4_ | 169.0880,125.0993 | Eucommiol |
| 27 | 10.09 | 171.1033 | C_9_H_16_O_3_ | 153.0938,127.1135,99.0843 | Deoxyeucommiol and its isomer |
| 28 | 11.34 | 171.1034 | C_9_H_16_O_3_ | 153.0922,127.1129,125.0982,99.0835 |  |
| 29 | 4.43 | 179.0352 | C_9_H_8_O_4_ | 135.0465 | Caffeic acid and its isomers |
| 30 | 10.29 | 179.0351 | C_9_H_8_O_4_ | 134.9897,105.0386,91.0000 |  |
| 31 | 6.22 | 179.0356 | C_9_H_8_O_4_ | 134.9902,109.0312 |  |
| 32 | 5.3 | 179.0355 | C_9_H_8_O_4_ | 135.0466 |  |
| 33 | 9.99 | 207.0665 | C_11_H_12_O_4_ | 179.0356 | Ethyl 3-(3,4-dihydroxyphenyl)acrylate and its isomer |
| 34 | 10.28 | 207.0666 | C_11_H_12_O_4_ | 179.0355,135.0464 |  |
| 35 | 6.37 | 193.0510 | C_10_H_10_O_4_ | 178.0290 | Pinusolidic acid and its isomer |
| 36 | 7.92 | 193.0507 | C_10_H_10_O_4_ | 178.0270 |  |
| 37 | 8.42 | 193.0510 | C_10_H_10_O_4_ | 178.0279,136.0180,149.0628 |  |
| 38 | 7.97 | 181.0508 | C_9_H_10_O_4_ | 108.0241 | 3,4-dihydroxyphenylpropionic acid |
| 39 | 3.54 | 353.0873 | C_16_H_18_O_9_ | 191.0569,173.0460 | Chlorogenic acid |
| 40 | 8.63 | 167.0388 | C_8_H_8_O_4_ | 96.0627 | Vanillic acid and its isomer |
| 41 | 4.49 | 167.0356 | C_8_H_8_O_4_ | 152.0131,123.0427,108.0243,91.0219 |  |
| 42 | 15.03 | 163.0407 | C_9_H_8_O_3_ | 229.1065,119.0527 | p-Coumaric acid and its isomer |
| 43 | 5.84 | 163.0404 | C_9_H_8_O_3_ | 119.0520,93.0370 |  |
| 44 | 9.36 | 285.0403 | C_15_H_10_O_6_ | 151.0051,133.0313 | Kaempferol |
| 45 | 6.49 | 593.1503 | C_27_H_30_O_15_ | 285.0413 | Kaempferol-3-rutinoside |
| 46 | 9.4 | 301.0353 | C_15_H_10_O_7_ | 178.9996,151.0043 | Quercetin |
| 47 | 18.5 | 289.0716 | C_15_H_14_O_6_ | 289.0724,177.0548 | Catechin |
| 48 | 6.19 | 609.1461 | C_27_H_30_O_16_ | 301.0352 | Rutinum |
| 49 | 6.5 | 447.0925 | C_21_H_20_O_11_ | 285.0409 | Quercitrin |
| 50 | 7.55 | 367.2115 | C_22_H_28_N_2_O_3_ | 349.2026,331.1922,305.2127 | Hirsutine |

**Supplementary Table S2 Summary of adverse events and adverse drug reactions classified by times and cases (ratio%)^a^ (SS analysis)**

|  | Test Group (n=30) | Control Group (n=29) | Total (n=59) |
| --- | --- | --- | --- |
|  | N (%) | N (%) | N (%) |
| AEs | 12, 12 (40) | 11, 10 (34.48) | 23, 22 (37.29) |
| ADRs | 8, 8 (26.67) | 7, 6 (20.69) | 15, 14 (23.73) |
| Serious AEs | 0 (0) | 0(0) | 0 (0) |
| Adverse events leading to drop-out | 0 (0) | 0(0) | 0 (0) |

*Note: ^a^ The percentage was calculated with the number of subjects in each group as the denominator.*

**Supplementary Table S3 Incidence of adverse events and adverse drug reactions (SS analysis)**

|  | Test Group (n=30) | Control Group (n=29) | *P* |
| --- | --- | --- | --- |
|  | N (%) | N (%) |  |
| AEs | 12 (40) | 10 (34.48) | 0.66 |
| ADRs | 8 (26.67) | 6 (20.69) | 0.59 |

**Supplementary Table S4 Details of adverse events**

|  | Drug NO. | Center NO. | Item | AE start date | AE end date | Severity of an AE | Serious AE | Action taken with study drug | Is the AE treated with symptomatic treatment | AE Outcome | Has emergency unblinding occurred in response to an AE | Has a patient withdrew from the trial because of an AE | Is an AE related to the drug |
| --- | --- | --- | --- | --- | --- | --- | --- | --- | --- | --- | --- | --- | --- |
| Control group | 2 | 1 | Elevated peripheral blood platelet count | 2019-04-01 | 2019-04-30 | Mild | No | The dose remained unchanged | No | No improvement | No | No | Not related |
|  | 12 | 1 | Elevated urinary β_2_m | 2019-10-15 | UK-UK-UK | Mild | No | The dose remained unchanged | No | Unknown | No | No | Possibly related |
|  | 14 | 1 | Urinary tract infection | 2019-10-17 | 2019-11-14 | Mild | No | The dose remained unchanged | No | Improvement | No | No | Possibly related |
|  | 20 | 1 | Urinary tract infection | 2019-12-24 | UK-UK-UK | Mild | No | Not applicable | No | Unknown | No | No | Not related |
|  | 24 | 2 | Elevated serum uric acid | 2019-12-15 | UK-UK-UK | Mild | No | The dose remained unchanged | No | Unknown | No | No | Possibly related |
|  | 33 | 2 | Renin was below normal | 2020-06-23 | UK-UK-UK | Mild | No | Not applicable | No | Unknown | No | No | Possibly related |
|  | 35 | 2 | Decreased hemoglobin level | 2020-07-29 | UK-UK-UK | Mild | No | Not applicable | No | Unknown | No | No | Possibly related |
|  | 53 | 3 | Hypoglycemia | 2020-06-11 | UK-UK-UK | Mild | No | The dose remained unchanged | Non-pharmacological therapy | No improvement | No | No | Not related |
|  | 56 | 3 | Hyperuricemia | 2020-06-11 | UK-UK-UK | Mild | No | The dose remained unchanged | No | No improvement | No | No | Possibly related |
|  | 56 | 3 | Hyperhomocysteinemia | 2020-06-11 | UK-UK-UK | Mild | No | The dose remained unchanged | No | No improvement | No | No | Possibly related |
|  | 60 | 3 | Possibility of urinary calculi | 2020-06-18 | UK-UK-UK | Mild | No | The dose remained unchanged | Non-pharmacological therapy | No improvement | No | No | Not related |
| Test group | 4 | 1 | High blood pressure | 2019-05-11 | 2019-05-14 | Mild | No | Trial drug interruption | No | Recovery | No | No | Not related |
|  | 9 | 1 | Hyperuricemia | 2019-10-27 | UK-UK-UK | Mild | No | Not applicable | No | Unknown | No | No | Possibly related |
|  | 11 | 1 | Elevated urinary β_2_m | 2019-11-12 | UK-UK-UK | Mild | No | Not applicable | No | Unknown | No | No | Possibly related |
|  | 16 | 1 | Elevated fasting glucose | 2019-11-26 | 2019-UK-UK | Mild | No | Not applicable | No | Recovery | No | No | Not related |
|  | 17 | 1 | Acute upper respiratory infections | 2019-11-22 | 2019-11-25 | Mild | No | The dose remained unchanged | Pharmacological therapy | Improvement | No | No | Not related |
|  | 19 | 1 | Elevated urinary β_2_m | 2019-11-11 | 2019-12-10 | Mild | No | The dose remained unchanged | No | No improvement | No | No | Possibly related |
|  | 21 | 2 | Insomnia | 2019-05-23 | 2019-05-24 | Mild | No | The dose remained unchanged | Pharmacological therapy | Recovery | No | No | Possibly related |
|  | 27 | 2 | Elevated serum glucose | 2020-01-15 | UK-UK-UK | Mild | No | Not applicable | No | Unknown | No | No | Possibly related |
|  | 37 | 2 | Elevated serum uric acid | 2020-08-03 | UK-UK-UK | Mild | No | Not applicable | No | Unknown | No | No | Possibly related |
|  | 48 | 3 | abnormal liver function | 2020-01-03 | UK-UK-UK | Mild | No | The dose remained unchanged | No | No improvement | No | No | Possibly related |
|  | 49 | 3 | Hyperuricemia | 2020-01-03 | UK-UK-UK | Mild | No | The dose remained unchanged | No | No improvement | No | No | Not related |
|  | 55 | 3 | Hyperhomocysteinemia | 2020-06-11 | UK-UK-UK | Mild | No | The dose remained unchanged | No | No improvement | No | No | Not related |

*Note: UK indicates that patients were lost to follow-up for the AEs.*

**Supplementary Table S5 Details of adverse drug reaction**

|  | Drug NO. | Center NO. | Item | AE start date | AE end date | Severity of an AE | Serious AE | Action taken with study drug | Is the ADR treated with symptomatic treatment | AE Outcome | Has emergency unblinding occurred in response to an AE | Has a patient withdrew from the trial because of an AE | Is an AE related to the drug |
| --- | --- | --- | --- | --- | --- | --- | --- | --- | --- | --- | --- | --- | --- |
| Test group | 9 | 1 | Hyperuricemia | 2019-10-27 | UK-UK-UK | Mild | No | Not applicable | No | Unknown | No | No | Possibly related |
|  | 11 | 1 | Elevated urinary β_2_m | 2019-11-12 | UK-UK-UK | Mild | No | Not applicable | No | Unknown | No | No | Possibly related |
|  | 19 | 1 | Elevated urinary β_2_m | 2019-11-11 | 2019-12-10 | Mild | No | The dose remained unchanged | No | No improvement | No | No | Possibly related |
|  | 21 | 2 | Insomnia | 2019-05-23 | 2019-05-24 | Mild | No | The dose remained unchanged | Pharmacological therapy | Recovery | No | No | Possibly related |
|  | 27 | 2 | Elevated serum glucose | 2020-01-15 | UK-UK-UK | Mild | No | Not applicable | No | Unknown | No | No | Possibly related |
|  | 37 | 2 | Elevated serum uric acid | 2020-08-03 | UK-UK-UK | Mild | No | Not applicable | No | Unknown | No | No | Possibly related |
|  | 48 | 3 | Abnormal liver function | 2020-01-03 | UK-UK-UK | Mild | No | The dose remained unchanged | No | No improvement | No | No | Possibly related |
|  | 55 | 3 | Hyperhomocysteinemia | 2020-06-11 | UK-UK-UK | Mild | No | The dose remained unchanged | No | No improvement | No | No | Possibly related |
| Control group | 12 | 1 | Elevated urinary β_2_m | 2019-10-15 | UK-UK-UK | Mild | No | The dose remained unchanged | No | Unknown | No | No | Possibly related |
|  | 14 | 1 | Urinary tract infection | 2019-10-17 | UK-UK-UK | Mild | No | The dose remained unchanged | No | Improvement | No | No | Possibly related |
|  | 24 | 2 | Elevated serum uric acid | 2019-12-15 | UK-UK-UK | Mild | No | The dose remained unchanged | No | Unknown | No | No | Possibly related |
|  | 33 | 2 | Renin was below normal | 2020-06-23 | UK-UK-UK | Mild | No | Not applicable | No | Unknown | No | No | Possibly related |
|  | 35 | 2 | Decreased hemoglobin level | 2020-07-29 | UK-UK-UK | Mild | No | Not applicable | No | Unknown | No | No | Possibly related |
|  | 56 | 3 | Hyperuricemia | 2020-06-11 | UK-UK-UK | Mild | No | The dose remained unchanged | No | No improvement | No | No | Possibly related |
|  | 56 | 3 | Hyperhomocysteinemia | 2020-06-11 | UK-UK-UK | Mild | No | The dose remained unchanged | No | No improvement | No | No | Possibly related |

*Note: UK indicates that patients were lost to follow-up for the AEs.*
